# Fluorescently Activated Cell Sorting Followed by Microarray Profiling of Helper T Cell Subtypes from Human Peripheral Blood

**DOI:** 10.1371/journal.pone.0111405

**Published:** 2014-11-07

**Authors:** Chiaki Ono, Zhiqian Yu, Yoshiyuki Kasahara, Yoshie Kikuchi, Naoto Ishii, Hiroaki Tomita

**Affiliations:** 1 Department of Disaster Psychiatry, Internal Research Institute of Disaster Science, Tohoku University, Sendai, Japan; 2 Department of Biological Psychiatry, Tohoku University Graduate School of Medicine, Sendai, Japan; 3 Department of Microbiology and Immunology, Tohoku University Graduate School of Medicine, Sendai, Japan; 4 Tohoku Medical Megabank Organization, Tohoku University, Sendai, Japan; Mie University Graduate School of Medicine, Japan

## Abstract

**Background:**

Peripheral blood samples have been subjected to comprehensive gene expression profiling to identify biomarkers for a wide range of diseases. However, blood samples include red blood cells, white blood cells, and platelets. White blood cells comprise polymorphonuclear leukocytes, monocytes, and various types of lymphocytes. Blood is not distinguishable, irrespective of whether the expression profiles reflect alterations in (a) gene expression patterns in each cell type or (b) the proportion of cell types in blood. CD4^+^ Th cells are classified into two functionally distinct subclasses, namely Th1 and Th2 cells, on the basis of the unique characteristics of their secreted cytokines and their roles in the immune system. Th1 and Th2 cells play an important role not only in the pathogenesis of human inflammatory, allergic, and autoimmune diseases, but also in diseases that are not considered to be immune or inflammatory disorders. However, analyses of minor cellular components such as CD4^+^ cell subpopulations have not been performed, partly because of the limited number of these cells in collected samples.

**Methodology/Principal Findings:**

We describe fluorescently activated cell sorting followed by microarray (FACS–array) technology as a useful experimental strategy for characterizing the expression profiles of specific immune cells in the circulation. We performed reproducible gene expression profiling of Th1 and Th2, respectively. Our data suggest that this procedure provides reliable information on the gene expression profiles of certain small immune cell populations. Moreover, our data suggest that GZMK, GZMH, EOMES, IGFBP3, and STOM may be novel markers for distinguishing Th1 cells from Th2 cells, whereas IL17RB and CNTNAP1 can be Th2-specific markers.

**Conclusions/Significance:**

Our approach may help in identifying aberrations and novel therapeutic or diagnostic targets for diseases that affect Th1 or Th2 responses and elucidating the involvement of a subpopulation of immune cells in some diseases.

## Introduction

Comprehensive gene expression analyses of peripheral blood samples have been performed to identify biomarkers for a wide range of diseases such as leukemia [1], [2], autoimmune diseases [3], [4], graft-versus-host disease [5], and inflammatory [6] and allergic disorders [7], [8], which primarily affect peripheral blood cells. Expression profiling of blood samples has also been applied to diseases that primarily affect the brain (e.g., demyelinating diseases [9], neurodegenerative diseases [10], [11], and psychiatric disorders [12], [13]) or peripheral organs other than blood (e.g., cancers [14], [15] and diabetes mellitus [16]). There are several reasons for researches to identify molecules dysregulated in peripheral blood samples from patients with these diseases primarily unrelated to peripheral blood. (1) Immune cells in the affected organ and peripheral blood interact. Dysregulated molecules in immune cells circulating in peripheral blood may directly or indirectly influence the pathogenesis in the affected organ or reflect immunological conditions related to the affected organ. (2) The affected organ and peripheral blood from the same individual share exactly the same genomic coding information and may therefore have similar transcriptional regulation patterns. A part of the dysregulated transcriptional activities in the affected organ can also be observed in peripheral blood in the same manner. (3) Blood samples are relatively easy to obtain compared to other organ tissues or cells.

In addition to the lack of complete knowledge about the mechanisms linking aberrations in peripheral blood with the pathogenesis of the affected organ, there is another limitation to comprehensive gene expression studies of peripheral blood samples. A blood sample comprises red blood cells, white blood cells, and platelets. White blood cells consist of polymorphonuclear leukocytes, monocytes, and various types of lymphocytes. Because blood samples utilized for gene expression studies are heterogeneous mixtures of various types of cells, it is difficult to determine with certainty of whether an expression profile reflects alterations in (a) gene expression patterns in each cell type or (b) the proportion of cell types in blood. Moreover, alterations in a gene expression pattern in a certain cell type can be offset by changes in the expression profiles of the other cell types in a blood sample. In this context, the expression profiles of major components of blood samples, such as CD11^+^ monocytes or CD4^+^ helper T (Th) cells, have been evaluated using magnetic cell separation [17], [18]. However, analyses of minor cellular components, such as CD4^+^ cell subpopulations, have not been performed in part because of the limited number of these cells in the collected samples.

To solve these problems, we developed a protocol of fluorescently activated cell sorting followed by microarray (FACS–array) suitable for characterizing the gene expression profiles of specific immune cells in blood samples. The FACS-array approach has been applied to various kinds of cells and tissues, including neuronal and blood cells [19], [20], [21]. Several studies used the FACS–array approach to isolate subpopulations of leukocytes, including CD4^+^ T cells, CD8^+^ T cells, B cells, monocytes, and granulocytes [21]. However, these subpopulations are themselves heterogeneous. For example, CD4^+^ T cells consist of subclasses, including type 1 and type 2 T helper cells (Th1 and Th2, respectively). The comprehensive gene expression profiling of these minor subpopulations of human blood cells remains unevaluated, probably due to technical difficulties with analysis of very small number of cells

In this study, we used the FACS–array procedure to characterize the expression profiles of type 1 and type 2 Th cells (Th1 and Th2, respectively). CD4^+^ Th cells are classified into two functionally distinct subclasses, Th1 and Th2 cells, on the basis of the unique characteristics of their secreted cytokines and roles in the immune system. Th1 cells synthesize interleukin-2 (IL-2), interferon-γ(IFN-γ), and tumor necrosis factor-β and induce a phagocytic cell-mediated immune response and proinflammatory effects, whereas Th2 cells synthesize IL-4, IL-5, IL-6, and IL-10 and induce a nonphagocytic humoral immune response and anti-inflammatory effects [22]. Th1 and Th2 cells have been shown to play an important role not only in the pathogenesis of human inflammatory, allergic, and autoimmune diseases [23], [24] but also in diseases that are not considered immune or inflammatory disorders [25], [26]. For example, the ratio of functional activities of Th2 cells to those of Th1 cells is elevated in blood samples from patients with schizophrenia [27], mood disorders [28], trauma-related mental health conditions [29], [30], and lupus erythematosus. This ratio is lowered in patients with rheumatoid arthritis [31]. In these previous studies, the Th1/Th2 imbalance was analyzed on the basis of the levels of serum cytokines, which are presumably secreted by Th1 and Th2 cells.

In contrast to this indirect evidence suggestive of dysfunction of Th1 and/or Th2 cells, our FACS–array procedure provides exact and comprehensive information on the molecules dysregulated in Th1 and Th2 cells in blood samples from patients, along with the exact ratio of Th1 and Th2 cell numbers. To date, there have been no studies aimed at evaluating the expression profiles of specific immune cells, except for microarray analysis of artificially differentiated Th1 and Th2 cells, which were derived from CD4^+^ cells *in vitro* by stimulation with IL-12 and IL-4, respectively [32]. Microarray studies of intrinsic Th1 and Th2 cells in blood samples should provide useful information for understanding the immune cell-relevant pathophysiology of various types of diseases. This article describes the FACS–array procedure as a useful experimental strategy that is useful for characterizing the expression profiles of specific immune cells from blood samples under unstimulated conditions. We here performed the reproducible gene expression profiling of Th1 and Th2 cells.

## Materials and Methods

### PBMC Isolation

Blood samples (20 ml) were drawn from 23 healthy individuals after obtaining written informed consent. Subjects who were affected by health conditions, including allergic or infectious diseases, or who took medication for such conditions were excluded from the study. To isolate peripheral blood mononuclear cells (PBMCs), each blood sample was diluted with an equal amount of phosphate-buffered saline (PBS; Gibco BRL/Invitrogen Technologies, Carlsbad, CA, USA) and overlaid onto Ficoll-Paque PLUS separation medium (GE Healthcare, Buckinghamshire, England). The cells at the plasma/Ficoll interface were collected and washed with PBS containing 10 mM EDTA and 2% fetal bovine serum (FBS), followed by a wash with RPMI 1640 medium containing 10% FBS. The total number of PBMCs was counted using a C-chip cell counter (NanoEnTek, Seoul, Korea). The cells were then cryopreserved at a concentration of approximately 1×10^7^ cells/mL in RPMI 1640 medium containing 10% FBS and 10% dimethyl sulfoxide (Wako, Osaka, Japan). Immediately after isolation, the cells were frozen at −80°C for 24 h. Subsequently, the cells were kept in liquid nitrogen until they were subjected to cell sorting. All experimental procedures described in this article were carried out according to a protocol approved by the Ethics Committee of Tohoku University Graduate School of Medicine.

### Antibodies and Flow Cytometry

**Figure 1:**
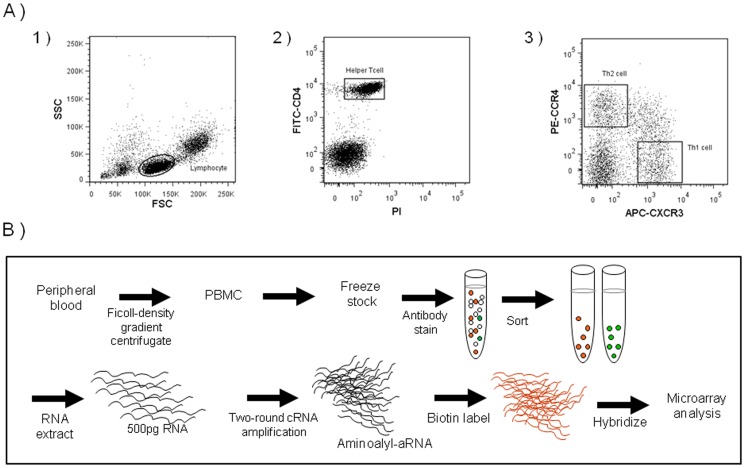
Schematic of the FACS–array procedure for peripheral blood cells. (**A**) Schematic of analysis of helper T (Th) cells using the FACS–array procedure for peripheral blood cells, where PBMCs are isolated on a density gradient centrifuge and stained with fluorescence-labeled antibodies (CD4-FITC, CXCR3-APC, and CCR4-PE) and PI for FACS. Total RNA was extracted from each subclass of lymphocytes and subjected to two rounds of cRNA amplification. Synthesized aminoallyl-aRNA samples were labeled with biotin and subjected to microarray analysis. (**B**) Dot plot imaging of FACS isolation of Th1 and Th2 cells. (1) Lymphocytic subpopulation of PBMCs was selected on the basis of their unique forward and side scatter properties on fluorocytometry. (**2**) Among the lymphocytes, PI-negative (viable) CD4^+^ Th cells were selected. (3) On the basis of the signal intensity of fluorostaining for the CXCR3 and CCR4 markers, the viable Th cells were separated into two subgroups: CXCR3^+^/CCR4^−^ as Th1 cells and CXCR3^−^/CCR4^+^ as Th2 cells.

The following monoclonal antibodies were used to isolate subpopulations of Th cells from human blood samples using a FACS system: FITC-conjugated anti-human CD4 antibody (clone PRA-T4, BD Biosciences Pharmingen, San Jose, CA, USA) for labeling CD4^+^ Th cells, APC-conjugated anti-human CXCR3 (CD183) antibody (clone 1C6, BD Biosciences Pharmingen) for labeling CXCR3^+^ Th1 cells, and PE-conjugated anti-human CCR4 antibody (clone 1G1, BD Biosciences Pharmingen) for labeling CCR4^+^ Th2 cells. A nonpermeating red fluorescent dye, propidium iodide (PI), was used to stain dead cells. To isolate Th1 and Th2 cells, the frozen PBMCs were thawed rapidly, washed with PBS, and stained with fluorophore-conjugated monoclonal antibodies specific to the surface markers, and separated on a FACS system, Aria (Becton, Dickinson and Company, Franklin Lakes, NJ, USA) as follows. (1) The lymphocytic subpopulation of PBMCs was selected on the basis of their unique forward and side scatter properties on fluorocytometry. (2) Among the lymphocytes, PI-negative (viable) cells were selected. (3) Among the viable lymphocytes, CD4^+^ Th cells were selected. (4) On the basis of the signal intensity of fluorostaining for the CXCR3 and CCR4 markers, viable Th cells were separated into four subgroups, CXCR3^+^/CCR4^−^ Th1 cells, CXCR3^−^/CCR4^+^ Th2 cells, CXCR3^+^/CCR4^+^ double-positive cells, and CXCR3^−^/CCR4^−^ double-negative cells. Data was analyzed using FACS Diva software (version 4.0.1.2; Becton, Dickinson and Company). (Figure 1A).

### RNA Isolation

Total RNA was isolated from each cell sample using the RNeasy Mini or Micro RNA isolation kit (Qiagen, Valencia, CA, USA). The RNeasy Mini kit was used with samples 1–6 in Table 1, which were collected from 2008/10/27 to 2008/12/24. The RNeasy kit was replaced with the Micro kit after 2009/2/19 to obtain highly concentrated RNA samples and applied to samples 7–23. Genomic DNA in the RNA samples was digested with RNase-free DNase I (Qiagen). The total RNA was eluted with 30 µl (from the RNeasy Mini columns) or 15 µl (from the RNeasy MinElute Spin Columns of the Micro kit) of RNase-free water. The concentration, 28S/18S ribosomal RNA (rRNA) ratio, and RNA integrity number (RIN) of the purified RNA samples were measured using the 2100 Bioanalyzer (Agilent, Santa Clara, CA, USA) with the RNA 6000 Pico Chip kit (Agilent). The RNA samples were then stored at −80°C until use.

**Table 1:** Characteristics of the samples and demographics of the subjects.

Sample ID	Gender	Age	Date	PBMCs (107)	% of CD4+				Th1				Th2				
					Th1	Th2	WP	WN	Cell count	Total RNA [pg]	rRNA ratio	RIN	Cell count	Total RNA [pg]	rRNA ratio	RIN	Exclusion
1	M	29	2008/10/27	2.3	13.4	12.8	4.0	60.0	135,950	39,046	1.05	6.6	125,129	28,099	2.58	9.8	
2	M	33	2008/10/27	2.4	2.7	10.1	0.2	79.6	27,508	181	0.00	0.0	118,257	19,591	2.14	9.6	○
3	M	45	2008/10/27	2.6	13.6	13	4.8	49.5	224,169	29,106	1.57	9.5	195,447	53,970	2.80	9.7	
4	M	35	2008/10/27	2.5	-	-	-	-	45,724	1,899	1.67	8.4	79,310	15,866	1.38	9.2	
5	F	24	2008/12/24	2.3	22.4	9.9	1.8	50.2	402,727	33,177	1.81	9.8	169,925	20,788	2.25	9.5	
6	M	31	2008/12/24	1.7	15.1	24.7	2.2	50.9	90,545	1,225	1.25	9.2	156,472	8,437	1.82	9.9	
7	M	29	2009/2/19	1.3	20.3	7.1	2.8	60.6	100,127	31,412	1.64	9.2	38,771	8,921	2.29	7.2	
8	F	25	2009/2/19	1.3	21.5	11.6	3.0	56.0	228,225	126,966	1.75	9.7	138,596	22,862	2.12	9.7	
9	F	40	2009/2/19	2.3	9.8	4.8	0.9	79.4	110,375	22,104	1.07	5.1	52,798	17,552	1.27	6.6	○
10	F	40	2009/2/19	2.4	23	7.1	3.0	59.8	300,216	253,100	1.76	9.7	103,144	38,941	2.06	9.4	
11	F	31	2009/2/25	2.3	13.9	15.6	2.1	52.8	137,250	60,538	2.03	9.7	180,248	112,817	2.58	9.9	
12	F	45	2009/2/25	3.5	21.3	15.4	3.1	46.7	277,014	140,393	2.44	9.9	202,758	70,557	1.34	9.7	
13	F	24	2009/2/25	2.9	7.4	6.0	1.1	80.3	329,185	228,191	2.12	9.8	311,759	109,503	2.41	9.9	
14	F	41	2009/3/18	1.5	29.5	12.9	8.2	39.0	471,293	79,309	0.85	6.8	256,889	43,138	1.58	9.0	○
15	F	58	2009/11/5	-	-	-	-	-	-	-	-	-	-	-	-	-	○
16	M	64	2009/11/5	1.5	9.2	10.6	6.4	57.3	68,056	8,820	1.60	9.0	235,539	53,617	1.64	8.7	
17	M	33	2009/12/8	1.5	4.8	13.2	0.6	72.9	53,624	22,593	0.00	2.7		18,036	2.41	8.1	○
18	F	42	2009/12/8	1.6	9.1	18.7	2.2	56.1	75,100	14,538	1.58	8.3	120,117	36,742	1.00	7.3	○
19	F	27	2009/12/8	2.3	4.3	14.6	0.8	69.7	48,775	15,117	1.40	3.5	136,106	22,727	1.69	9.6	○
20	F	61	2009/12/8	1.9	20.7	12.8	13.5	36.4	100,929	3,452	0.00	2.4	145,623	22,277	1.60	8.8	○
21	M	43	2009/12/8	2.8	34.3	17.1	8.8	24.9	120,054	3,215	0.00	1.8	90,503	24,263	1.16	7.3	○
22	F	58	2009/12/8	1.8	17.8	13.3	4.2	57.2	73,052	19,272	1.68	6.6	127,457	34,239	1.21	7.1	
23	M	47	2009/12/15	2.2	17.2	6.9	4.1	61.0	39,779	2,778	0.20	5.4	68,117	7,609	1.70	9.3	○
	Av	39.4		2.1	15.8	12.3	3.7	57.2	157,258	51,656	1.25	7.3	143,378	35,934	1.86	8.9	
	SD	12.0		0.6	8.3	4.7	3.2	14.2	126,384	72,241	0.76	2.8	67,308	29,113	0.53	1.1	

The table shows gender and age, the number of PBMCs from each subject, the cell count and extracted RNA quality indicators of each sorted type 1 and type 2 helper T (Th1 and Th2, respectively) cell sample, and the proportion of each subclass among the CD4^+^ cells. As the proportion of each subclass among the CD4^+^ cells, percentages of Th1, Th2, CXCR3^+^/CCR4^+^ double-positive population (WP), and CXCR3^−^/CCR4^−^ double-negative population (WN) in the CD4^+^ Th cells are shown in “% of CD4^+^” columns. RNA quality of each Th1 and Th2 sample is indicated as a 28S/18S ribosomal RNA (rRNA) ratio and an RNA integrity number (RIN) of total RNA measured using the Agilent Bioanalyzer 2100. The “Exclusion” column indicates whether a sample met the exclusion criteria: 28S/18S rRNA ratio of <1.0 or RIN of <6.5.

### Evaluation of the Effects of Freezing of Cells on the Quantity and Quality of RNA Extracted from PBMCs

Each PBMC sample was divided in two, and one of these PBMC aliquots was immediately subjected to 0.4% trypan blue staining and total RNA extraction procedures, as described above. The remaining half of the PBMC aliquots was stored in liquid nitrogen, as described above, for 4 days and subjected to 0.4% trypan blue staining and total RNA extraction. In both cases, the RNA samples were frozen at −80°C immediately after extraction. The quality and quantity of the RNA samples were measured using the Agilent Bioanalyzer 2100, as described above.

### Evaluation of Amplification Systems with Small Amounts of RNA for Microarray Analysis

There are at least three commercially available kits for amplifying several hundred picograms of total RNA to a certain amount of biotinylated cRNA that would be sufficient for the Illumina BeadChip–based microarray experiments: (1) TargetAmp 2-Round Aminoallyl aRNA amplification kit 1.0 (Epicentre Biotechnologies, Madison, WI, USA) with biotin-X-X-NHS (Epicentre Biotechnologies), (2) MessageAmp II aRNA amplification kit with biotin-11-CTP and biotin-16-UTP (Ambion, Austin, TX, USA), and (3) WT-Ovation FFPE RNA amplification system V2 (NuGEN, San Carlos, CA, USA). Six small aliquots (100 pg) of total RNA samples extracted from human peripheral blood leukocytes (Clontech Laboratories, Palo Alto, CA, USA) were amplified and biotinylated using each of the three kits in duplicates and applied to the Illumina Human-6v2 Expression BeadChips (Illumina, San Diego, CA, USA), according to the manufacturer's instructions. As a control, two standard amounts of aliquots (500 ng) of the same total RNA samples were subjected to biotinylated cRNA synthesis in duplicates following the standard protocol using the Illumina TotalPrep RNA amplification kit (Ambion) and applied to the same BeadChip microarray. We assessed the amplification efficiency of each kit, congruence between the duplicated microarray data, and linearity of the small amount- and standard amount-based microarray data.

### Microarray Analysis of Th1 and Th2 Cells

Total RNA (300–500 pg) extracted from each isolated cell sample was subjected to aminoallyl-aRNA amplification using the TargetAmp Round Aminoallyl aRNA amplification kit 1.0 and the MinElute cleanup kit (Qiagen). The aminoallyl-aRNA was labeled with biotin-X-X-NHS (Epicentre Biotechnologies) and processed again using the MinElute cleanup kit (Qiagen). After determination of the concentration using NanoDrop 2000 (Thermo Fisher Scientific, Waltham, MA, USA), 1.0–1.5 µg of biotin-labeled aRNA was hybridized to the Illumina Human-6v2 Expression BeadChips, according to the manufacturer's instructions. Each BeadChip was labeled with streptavidin–Cy3 (GE Healthcare), washed, and scanned on the Illumina Bead Station 500X (Illumina). Signal intensity of each BeadChip was measured and normalized using BeadStudio software (Illumina).

### Expression data

RNA expression data have been submitted to the Gene Expression Omnibus (GEO; http://www.ncbi.nlm.nih.gov/geo) with the series accession number GSE59295.

### Flow Cytometric validation of Th1- and Th2-specific genes detected in FACS–array experiments

Some of the genes detected as Th1-dominant genes in Th1 and Th2 microarray data are known to be expressed in natural killer (NK) cells. Therefore, we performed flow cytometric analysis with PE-labeled anti-human CD56 antibody. Similarly, we analyzed the expression of IL17RB, detected among Th2-dominant genes in the microarray data, using APC–Cy7-labeled anti-human IL17RB antibody in a flow cytometric experiment.

### Immunohistochemical validation of Th1- and Th2-Dominant genes detected in FACS–array experiments

To confirm the validity of the FACS–array procedure, representative Th1- and Th2-specific genes were selected from the top five on the basis of the microarray data, and coexpression of the protein product with the Th1-specific transcription factor T-bet and the Th2-specific transcription factor GATA3 was analyzed in human CD4^+^ T cells. Human CD14^−^/CD4^+^ Th cells were isolated from PBMCs using the CD14 and CD4 microbeads (Miltenyi Biotec, Bergisch Gladbach, Germany) and magnetic-activated cell sorting column technology (Miltenyi Biotec). Subsequently, the cells were spread on BioCoat collagen I 8-well culture slides (Becton, Dickinson and Company). The cells were incubated at room temperature for 20 min and fixed with 4% paraformaldehyde. After overnight incubation at 4°C with primary antibodies, the cells were rinsed in PBS and then incubated at room temperature in the dark with secondary antibodies. The primary antibodies used for immunostaining were as follows: anti-human T-bet (1∶200 dilution, RabMAb), anti-human GATA3 (1∶100 dilution, Novus Biologicals or Abcam), anti-Tbr-2 (EOMES; 1∶100 dilution, Millipore), anti-IL17RB (1∶100 dilution, Sigma), and anti-CNTNAP1 (1∶100 dilution, Sigma-Aldrich). The corresponding secondary antibodies were as follows: Alexa Fluor 488 or 594 -conjugated anti-rabbit IgG (1∶500 dilution, Invitrogen) for anti-Tbet; Alexa Fluor 488-conjugated anti-rabbit or mouse IgG (1∶500 dilution, Invitrogen) for anti-CNTNAP1; Alexa Fluor 594- conjugated anti-rabbit or mouse IgG (1∶500 dilution, Invitrogen) for anti-GATA3; and Alexa Fluor 488-conjugated anti-chicken IgG (1∶500 dilution, Invitrogen) for anti-Tbr2. After incubation with the secondary antibodies, the tissues were rinsed in PBS and subjected to nuclear staining with 4,6-diamidino-2-phenylindole (Invitrogen). Microscopic images were captured using a BIOREVO Bz9000 (KEYENCE, Osaka, Japan).

The numbers of cells that co-expressed EOMES and T-bet among EOMES-positive cells and among T-bet-positive cells were counted and used to calculate specificity and sensitivity of EOMES as a Th1 cell marker. Likewise, the numbers of cells that co-expressed CNTNAP1 and GATA-3 among CNTNAP1-positive cells and among GATA-3-positive cells were counted and used to calculate specificity and sensitivity of CNTNAP1 as a Th2 cell marker.

## Results

### FACS and RNA Extraction

Of 2.1±0.6 (mean ± SD) ×10^7^ PBMCs, 13.6±5.5% were PI^−^/CD4^+^ cells, which are thought to be viable Th cells. Among the PI^−^/CD4^+^ cells, 15.8±8.3% were CXCR3^+^/CCR4^−^ cells, which are believed to be Th1 cells, whereas 12.3±4.7% were CXCR3^−^/CCR4^+^ cells, which are thought to be Th2 cells. More than half of the PI^−^/CD4^+^ cells (57.2±14.2%) were CXCR3^−^/CCR4^−^ double-negative cells and 3.7±3.2% were CXCR3^+^/CCR4^+^ double-positive cells. The ratio of the Th1 cell number to the Th2 cell number varied widely among individuals (1.1±0.8).

The amount of total RNA extracted from 10^4^ each of the Th1 and Th2 cells was 0.07–8.43 ng and 0.54–6.3 ng, respectively (0.01–15.3 ng and 0.46–6.7 ng, respectively, from 1 ml of whole blood samples). The 28S/18S rRNA ratio of the Th1 and Th2 samples was 0.0–2.4 and 1.0–2.8, respectively, and RIN of the samples was 0–9.9 and 6.6–9.9, respectively. The samples with a 28S/18S rRNA ratio of <1.0 or RIN of <6.5 were excluded from further analysis (Table 1). Cell viability, calculated as a cell count per milliliter, after the freeze–thaw procedure was approximately 88%. There was no decrease in quantity or quality of RNA samples extracted from frozen stocks of PBMCs compared with fresh PBMCs.

### Evaluation of RNA Amplification Systems

**Figure 2:**
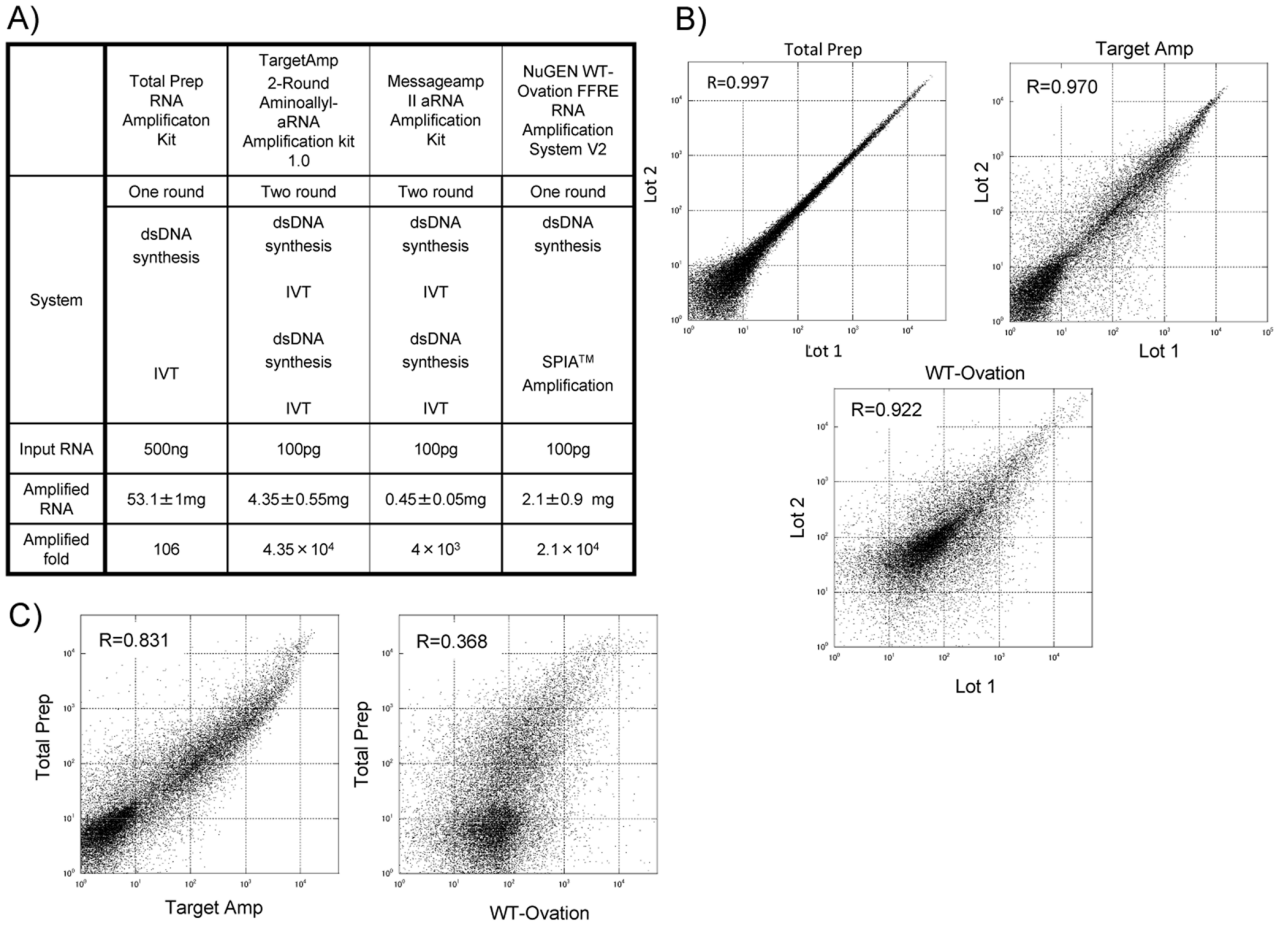
Evaluation of RNA amplification systems for the FACS-array procedure. (**A**) A summary of principles of the four RNA amplification systems and efficacy of amplification. Some data are shown as mean ± SD. (**B**) Scatter plots showing correlations between the gene expression profiles of duplicated batches of amplified RNA from the same small amount of human lymphocyte RNA using three RNA amplification systems. TotalPrep: amplified using the Total Prep RNA amplification kit (Illumina), Target Amp: amplified using the TargetAmp 2-round aminoallyl-aRNA amplification kit 1.0, and WT-Ovation: amplified using the WT-Ovation FFRE RNA amplification system V2 (NuGEN). (**C**) Scatter plots showing correlations between the gene expression profiles of amplified RNA from the same small amount of human lymphocyte RNA using TargetAmp/WT-Ovation and conventional TotalPrep.

The TargetAmp aRNA amplification kit, MessageAmp II aRNA amplification kit, and WT-Ovation FFPE RNA amplification kit amplified 100 pg of human leukocyte total RNA to 4.4±0.6 µg (43,500-fold), 0.45±0.05 µg (4500-fold), and 2.1±0.9 µg (21,000-fold) of biotinylated aRNA, respectively. The amplification efficiency of each of these three systems was much higher than that of the standard amplification protocol using the Illumina TotalPrep RNA amplification kit, which amplified 500 ng of the same total RNA to 53 µg of biotinylated cRNA (106-fold; Figure 2A). Because the amount of aRNA obtained using the MessageAmp II kit was not sufficient for a microarray experiment, biotinylated aRNA samples obtained using the TargetAmp and WT-Ovation kits were used in the Illumina Human-6v2 Expression BeadChip-based microarray experiment. TargetAmp kit-amplified human leukocyte total RNA (100 pg each) showed 8044 and 8260 transcripts in duplicated measurements with signal intensities of >50 and a detection *p* value of <0.05, among 48,701 transcripts. WT-Ovation kit-amplified human leukocyte total RNA samples showed 9306 and 9168 transcripts, which met the same criteria. These numbers were comparable to the number of transcripts, 9270 and 9393 observed in microarray data of the standard TotalPrep protocol-amplified human leukocyte total RNA samples.

Spearman's rank correlation coefficient of the duplicated microarray expression profiles of the samples amplified using the TargetAmp and MessageAmp II kits were 0.95 and 0.92, respectively, whereas this coefficient for the samples amplified using the standard TotalPrep kit was approximately 1.00 (Figure 2B). Spearman's correlation between the expression profiles of the samples amplified using the TargetAmp kit and standard TotalPrep protocol was 0.83, whereas that between the expression profiles of the samples amplified using the WT-Ovation kit and standard TotalPrep protocol was 0.37 (Figure 2C). The above data suggest that the TargetAmp kit yielded better reproducibility and linearity than the WT-Ovation kit. Accordingly, the TargetAmp kit was used for the rest of the FACS–array experiments.

### Overview of Th1–Th2 Microarray Expression Profiles

**Figure 3:**
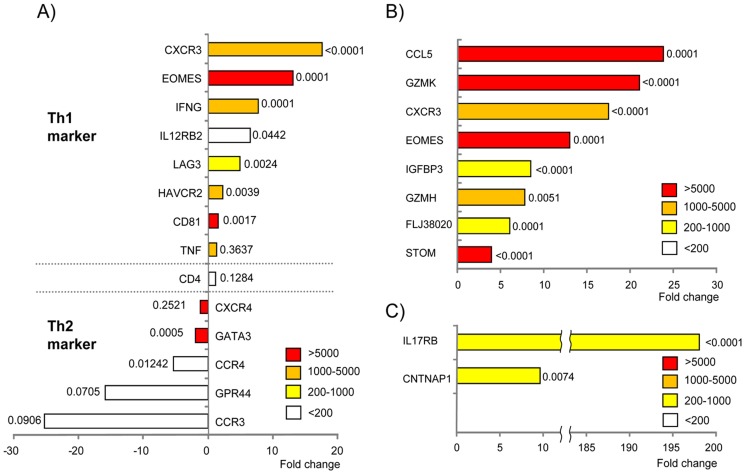
Differences in expression levels of representative genes between human Th1 and Th2 cells. Gene expression profiles of human Th1 and Th2 cells isolated from blood samples of 12 healthy individuals were analyzed using Illumina Human-6v2 Expression BeadChips arrays. Each bar represents a fold change of averaged signal intensity each gene in the Th1 microarray data divided by averaged signal intensity of the same gene in Th2 microarray data. Indicated next to each bar diagram is the *p* value obtained from a paired *t* test to evaluate a difference in signal intensity for each gene between the Th1 and Th2 microarray data from the 12 donors. Colors indicate the signal intensity of genes. Red: a very high expression level (>5000), orange: high level of expression (1000–5000), yellow: medium expression level (200–1000), and white: low expression level (<200). (**A**) Fold changes of well-established Th1 and Th2 genes in the comparison between human Th1 and Th2 microarray data. Positive fold changes mean that transcripts are more abundant in Th1 microarray data than in Th2 data, and negative values indicate the opposite. (**B**) Fold changes of the most prominent Th1-specific genes, which showed a signal intensity of>200 and a fold change of>2 in Th1 microarray data compared with that in Th2 data. (**C**) Fold changes of the most prominent Th2-specific genes, which showed a signal intensity of>200 and a fold change of>2 in Th2 microarray data compared with that in Th1 data.

Spearman's correlation of signal intensities of total probes between individuals among the 13 Th1 and Th2 aRNA samples was higher than 0.89 and 0.87, respectively, except for 1 outlier sample (Sample 3). Among 48,701 transcripts, 5891 transcripts passed the criteria (signal intensity, >50; detection *p* value, <0.05) in all 12 Th1 microarray data, whereas 5704 transcripts passed the same criteria in all 12 Th2 microarray data, and 5351 transcripts were overlapped between the Th1 and Th2 data sets. Signal intensities of representative Th1- and Th2-specific cytokines are shown in Figure 3A.

The Th1 marker *CXCR3* was expressed in all Th1 samples, and the average signal intensity of *CXCR3* was 17.4-fold higher in the Th1 samples than in the Th2 samples. The average signal intensity of other Th1 markers, *IFNG* and *IL12RB*, in the Th1 samples was 7.5- and 6.3-fold higher than that in the Th2 samples, respectively. On the other hand, the average signal intensity of other Th2 markers, *CCR4*, *GPR44 (CRTH2)*, and *CCR3*, in the Th2 samples was 5.2-, 15.8-, and 24.6-fold higher than that in the Th1 samples, respectively. (Figure 3A).

As novel candidate Th1-specific marker genes, 82 transcripts met the criteria (average signal intensity of >200 in the Th1 samples and >2-fold higher average signal intensity in the Th1 samples than in the Th2 samples analyzed in this study), which are shown in Table S1. Among them, eight genes (*CCL5*, *GZMK*, *CXCR3*, *EOMES*, *IGFBP3*, *GZMH* and *STOM*) showed >2-fold higher signal intensity in the Th1 samples than in the Th2 samples for all individuals (Figure 3A). As for candidate Th2-specific marker genes, 38 transcripts met the criteria (average signal intensity of >200 in the Th2 samples and >2-fold higher average signal intensity in the Th2 samples than in the Th1 samples analyzed in this study), which are shown in Table S2. Among them, two genes [*IL17RB* and (*contactin-associated protein 1 CNTNAP1*] showed >2-fold higher signal intensity in the Th2 samples than in the Th1 samples for all individuals (Figure 3C). Over-representation analysis indicated that genes relevant to the “disulfide bond” category (*p* = 7.61E−05, *p* = 0.012 after Bonferroni correction) and “zymogen” category (*p* = 1.23E−04, *p* = 0.018 after Bonferroni correction) were significantly overrepresented among the 82 Th1-dominant genes, whereas no specific gene category was overrepresented among the 38 Th2-dominant genes (Figure S1).

### Immunohistochemical Validation of Th1- and Th2-Dominant Genes Detected in FACS–array Experiments

**Figure 4:**
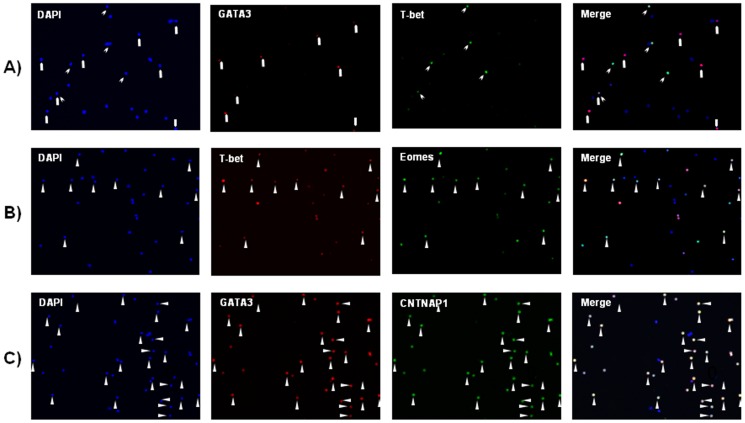
Coexpression of T-bet, GATA-3, and Th1- and Th2-dominant markers in CD4^+^ cells. (**A**) Costaining of CD4^+^ cells with antibodies to the Th1-specific transcription factor T-bet (red) and the Th2-specific transcription factor GATA-3 (green) indicated that T-bet^+^ cells and GATA-3^+^ cells were distinct cell populations among the CD4^+^ cells. Cell nuclei were stained with 4,6-diamidino-2-phenylindole (DAPI) (blue). (**B**) Costaining of CD4^+^ T cells with antibodies to T-bet (red) and EOMES (green) showed that T-bet and EOMES were coexpressed in the CD4^+^ cells. Cell nuclei were stained with DAPI (blue). (**C**) Costaining of CD4^+^ cells with antibodies to GATA-3 (red) and CNTNAP1 (green) showed that GATA-3 and CNTNAP1 were coexpressed in the CD4^+^ cells. Cell nuclei were stained with DAPI (blue).

To evaluate cell type-specific expression at the protein level, we performed immunohistochemical analysis of the protein expression of the novel candidate Th1 and Th2 markers and the coexpression of EOMES, IL17RB, and CNTNAP1 with the Th1-specific transcription factor T-bet (TBX21) and the Th2-specific transcription factor GATA3 (Figure 4A). After confirmation of distinct expression patterns between T-bet and GATA3 (Figure 4A), the coexpression of EOMES with T-bet (Figure 4B) and the coexpression of CNTNAP1 with GATA3 (Figure 4C) were observed among human CD4^+^ cells. Immunostaining with two specific antibodies to IL17RB failed to produce a signal in any of the human CD4^+^ cells, which suggests the expression of *IL17RB* mRNA, but not protein, in Th2 cells (data not shown).

The number of total EOMES-positive cells, total T-bet-positive cells, and cells that co-expressed EOMES and T-bet were 103, 93, and 75, respectively. The number of total CNTNAP1-positive cells, total GATA-3-positive cells, and cells that co-expressed CNTNAP1 and GATA-3 were 234, 236, and 234, respectively. Accordingly, the specificity and sensitivity of EOMES as a Th1 cell marker were 73% and 87.2%, respectively, whereas the specificity and sensitivity of CNTNAP1 as a Th2 cell marker were 100% and 99.2%, respectively.

## Discussion

Here we established a FACS–array protocol for assessing the gene expression profiles of minor cellular components of blood samples by validating each of the following processes (Figure 1B).

### Validity of RNA Extraction Procedures

The amount of RNA in the Th1 (0.26±0.26 pg/cell) and Th2 cells (0.24±0.12 pg/cell) was comparable to that extracted from leukocytes (0.25 pg/cell) in the Biology Data Book [33]. According to the book, the amount of RNA extracted from leukocytes is smaller than that extracted from other tissues, such as liver (2.5 pg/cell), kidney (1.1 pg/cell), and brain (2.6 pg/cell). These data suggest that the gene expression profiling of small populations of leukocytes is difficult. RIN, a widely accepted RNA quality indicator, of the Th1 and Th2 samples was 7.3±2.8 and 8.9±1.1, respectively. The 28S/18S rRNA ratio of the Th1 and Th2 samples was 1.2±0.8 and 1.9±0.5, respectively. Although most of the cells showed high RIN, 34.8% and 8.7% of the Th1 and Th2 cells, respectively, showed RIN of <6.5 or a 28S/18S rRNA ratio of <1.0. Low RIN may be because of damage caused to cells during sorting (Table 1). Our gene expression profile-based evaluation suggested that samples with RIN of <6.5 or a 28S/18S rRNA ratio of <1.0 should be excluded from the subsequent experiments, an approach that is consistent with previous reports [34].

After exclusion of these samples, the correlation coefficient of any combinations of the remaining samples was >0.95 and inclusion/exclusion of further samples did not significantly affect the gene expression profiling data.

### Evaluation of the Amplification System

The amount of total RNA extracted from Th1 and Th2 cells is approximately 30–50 ng/10 ml of a blood sample. On the other hand, the widely used Affymetrix microarray system requires 10 µg of total RNA (www.affymetrix.com), whereas even Illumina BeadChip system, which is one of the microarray systems that uses small amounts of RNA, recommends at least 500 ng of RNA. Therefore, to evaluate the gene expression profiles of minor cellular components, it is essential to amplify total RNA. To date, at least three RNA amplification systems are commercially available: TargetAmp, MessageAmp II, and WT-Ovation. In our study using 100 pg of high-quality total RNA from human lymphocytes, the TargetAmp and WT-Ovation kits, but not the MessageAmp II kit, produced the amount of amplified RNA sufficient for Illumina-based microarray experiments. According to the instruction manuals or comments from technical support of each vendor, the minimum required starting amount of total RNA samples for the TargetAmp or WT-Ovation kit is 10 pg. Nevertheless, the minimum amount for the MessageAmp II kit is 100 pg, which suggests that the first two systems above are suitable for smaller amounts of RNA samples.

The correlation coefficient of the duplicated microarray expression profiles of the samples amplified using the TargetAmp kit was 0.97, and the correlation of gene expression profiles developed using TargetAmp two round-amplified RNA and the standard one round-amplified (TotalPrep) RNA was 0.83. The correlation coefficient of duplicates of WT-Ovation kit-amplified DNA was 0.92. On the other hand, the correlation of gene expression profiles developed using WT-Ovation kit-amplified DNA and the standard one round-amplified RNA was only 0.37, which suggests that TargetAmp is the most reliable two-round amplification system (Figure 2C). The correlation of gene expression profiles developed using TargetAmp and the standard method was higher than that of gene expression profiles developed using WT-Ovation and the standard method. This may indicate that the mode of action of WT-Ovation is different from that of TargetAmp and the standard method, both of which are based on *in vitro* transcription amplification (Figure 2A). WT-Ovation is based on single primer isothermal amplification.

### Validity of the FACS–array Procedure, Which is Based on the Measurement of Established Th1 and Th2 Cell Markers

Among Th1 markers, *CXCR3* and *IFNG* showed signal intensities of >1000. On the other hand, the signal intensities of *IL-2* and *T-bet* were below reliably detectable levels in Th1 microarray data, although TBX21 protein expression in CD4^+^ cells was confirmed using immunohistochemistry (Figure 4). Both of the microarray-detectable genes, *CXCR3* and *IFNG*, showed 5.7- to 139.5-fold and 1.7- to 83.6-fold higher signal intensity, respectively, in Th1 cells than in Th2 cells from the same individuals.

Th2 markers *CCR3*, *CCR4*, and *GPR44* (*CRTH2*) were exclusively expressed in the Th2 cells, while these genes were not expressed in the Th1 cells. Signal intensities of the other major Th2 cytokines, *IL-4*, *IL-5*, and *IL-13*, were below reliably detectable levels in microarray data. Taken together, our data suggest that the FACS–array procedure provides reliable information on the gene expression profiles of certain small populations of immune cells.

Caution is needed when interpreting previous reports indicating that CCR4 is expressed not only on Th2 cells but also on CD25^+^/CD4^+^/FOXP3^+^ regulatory T cells (T_reg_ cells) [35], [36]. Although the population of CD4^+^/CXCR3^−^/CCR4^+^ cells constitutes 12.3±4.7% among CD4^+^ cells in the present study, previous studies indicated that the proportion of CCR4^+^/CD25^+^/CD4^+^/FOXP3^+^ T_reg_ cells is up to 5% among peripheral CD4^+^ cells [37]. In addition, average signal intensity for Gata-3 staining of CD4^+^/CXCR3^−^/CCR4^+^ cells is 6550, whereas average signal intensity for FOXP3 is 40. Thus, CD4^+^/CXCR3^−^/CCR4^+^ cells in the present study may include T_reg_ cells to some extent, but the majority of this CD4^+^CXCR3^−^CCR4^+^ population is likely to be Th2 cells.

### Novel Th1 and Th2 Marker Genes

In this study, *CCL5*, *GZMK*, *CXCR3*, *EOMES*, *IGFBP3*, *GZMH*, and *STOM* were found to be the most prominent marker genes for distinguishing Th1 cells from Th2 cells. Among them, *CCL5* and *CXCR3* are well-established Th1-specific markers [38], [39]. *EOMES* was recently recognized as a transcription factor that participates in T cell differentiation into Th1 cells [40]. Our data show sustained *EOMES* expression in mature Th1 cells, suggesting that it could serve as a Th1 cell marker in blood samples. Previous reports have indicated that human granzymes, including *GZMK* and *GZMH*, are predominantly expressed in NK cells and CD8^+^ T cells, whereas the expression levels of these genes are low in CD4^+^ T cells [41], [42]. In our study, in addition to granzymes, NK cell-related genes, *LRG1* and *NKG1*, were exclusively expressed in Th1 cells (Table S1). The Th1-specific transcription factor T-bet, as well as IFN-γ, are also expressed in NK cells [43], which supports the notion that among CD4^+^ T cells, Th1 cells may have gene expression profiles similar to those of NK cells. To our knowledge, previous findings never linked the expression of *IGFBP3*, and *STOM* with Th1 cells, and our data suggest that these molecules are novel Th1 cell markers.

Our data also indicate that two genes, *IL17RB* and *CNTNAP1*, are Th2 cell markers. IL17RB is the receptor for IL-17B and IL-17E (IL-25), which has been shown to induce Th2 responses. Recent studies revealed that IL17RB is highly expressed on the surface of a subset of naive and activated CD4^+^ invariant NK T cells. In addition, among CD4^+^ T cell subpopulations, IL17RB is expressed in activated Th2 central memory cells but not in activated T cells [44], [45], [46]. Our data show that IL17RB mRNA, but not protein, is expressed in mature Th2 cells. CNTNAP1 is known as neurexin IV. Neurexins are a large family of proteins that act as neuronal cell surface receptors. The function and localization of neurexins, however, have not been elucidated in immune cells. To our knowledge, CNTNAP1 has never been shown to be involved in Th2-specific cellular functions.

To date, Th1 and Th2 cells have been identified using a small number of cell surface markers. There should be heterogeneity among the Th1 and Th2 cells; therefore, there may be additional markers that can be used to identify Th1 and Th2 cell subpopulations. Our data indicate that there are genes that show more pronounced Th1- and Th2-specific gene expression patterns than the established markers. These markers should be tested for the detection of Th1 and Th2 cells or for the classification of T cell populations.

Among them, EOMES and CNTNAP1 were subjected to the protein expression analysis along with T-bet and GATA3 as Th1- and Th2-specific markers, respectively. At the beginning, we decided to validate the findings using cell sorting analysis of CD4^+^ cells double-stained with antibodies to EOMES and T-bet (or antibodies to CNTNAP1 and GATA-3); however, we could not detect specific signals of these double-stained cells probably because of difficulties with sorting of cells labeled with nuclear protein-specific markers. Therefore, we utilized the immunostaining approach, which is a well-established method for the analysis of nuclear proteins to confirm the coexpression of EOMES and T-bet (or CNTNAP1 and GATA-3). Our data suggest that EOMES and CNTNAP1 may be candidate markers for Th1 and Th2 cells, respectively, at least in immunostaining experiments.

In summary, we developed a FACS–array protocol to characterize the gene expression profiles of specific immune cells in blood samples and successfully applied this protocol to the characterization of the expression profiles of Th1 and Th2 cell populations. Our approach may help to identify aberrations and novel therapeutic or diagnostic targets for the diseases that affect Th1 or Th2 responses.

## Supporting Information

Figure S1 **Fold change of Th1-dominant genes which belong to significantly over-represented functional categories.** Over-representation analysis of Th1 dominant genes indicated that genes relevant to the “disulfide bond” category (*p* = 7.61E−05, *p* = 0.012 after Bonferroni correction) and “zymogen” category (*p* = 1.23E−04, *p* = 0.018 after Bonferroni correction) were significantly overrepresented. Figure S1 A and B indicate fold changed of Th1 which belong to “disulfide bond” and “zymogen” categories respectively. Th1 cell dominant genes Each bar represents a fold change of averaged signal intensity each gene in the Th1 microarray data divided by averaged signal intensity of the same gene in Th2 microarray data. Colors indicate the signal intensity of genes. Red: a very high expression level (>5000), orange: high level of expression (1000–5000), yellow: medium expression level (200–1000).
(TIF)

Table S1 **Th1-dominant genes.** These genes met the following criteria: average signal intensity of >200 in Th1 samples, signal intensity is >2-fold higher in Th1 samples than in Th2 samples, and the *p* value of paired Student's *t* test is <0.05 in the comparison of signal intensity between the Th1 and Th2 cells.
(XLSX)

Table S2 **Th2-dominant genes.** These genes met the following criteria: average signal intensity of >200 in Th2 samples, signal intensity is >2-fold higher in Th2 cells than in Th1 cells, and the *p* value of paired Student's t test is <0.05 in the comparison of signal intensity between the Th1 and Th2 cells.
(XLSX)
