# Supplementary figures and images for: Fluorescently Activated Cell Sorting Followed by Microarray Profiling of Helper T Cell Subtypes from Human Peripheral Blood
Source: PLoS One. 2014 Nov 7;9(11):e111405. doi: 10.1371/journal.pone.0111405 (PMC4224392; doi:10.1371/journal.pone.0111405)

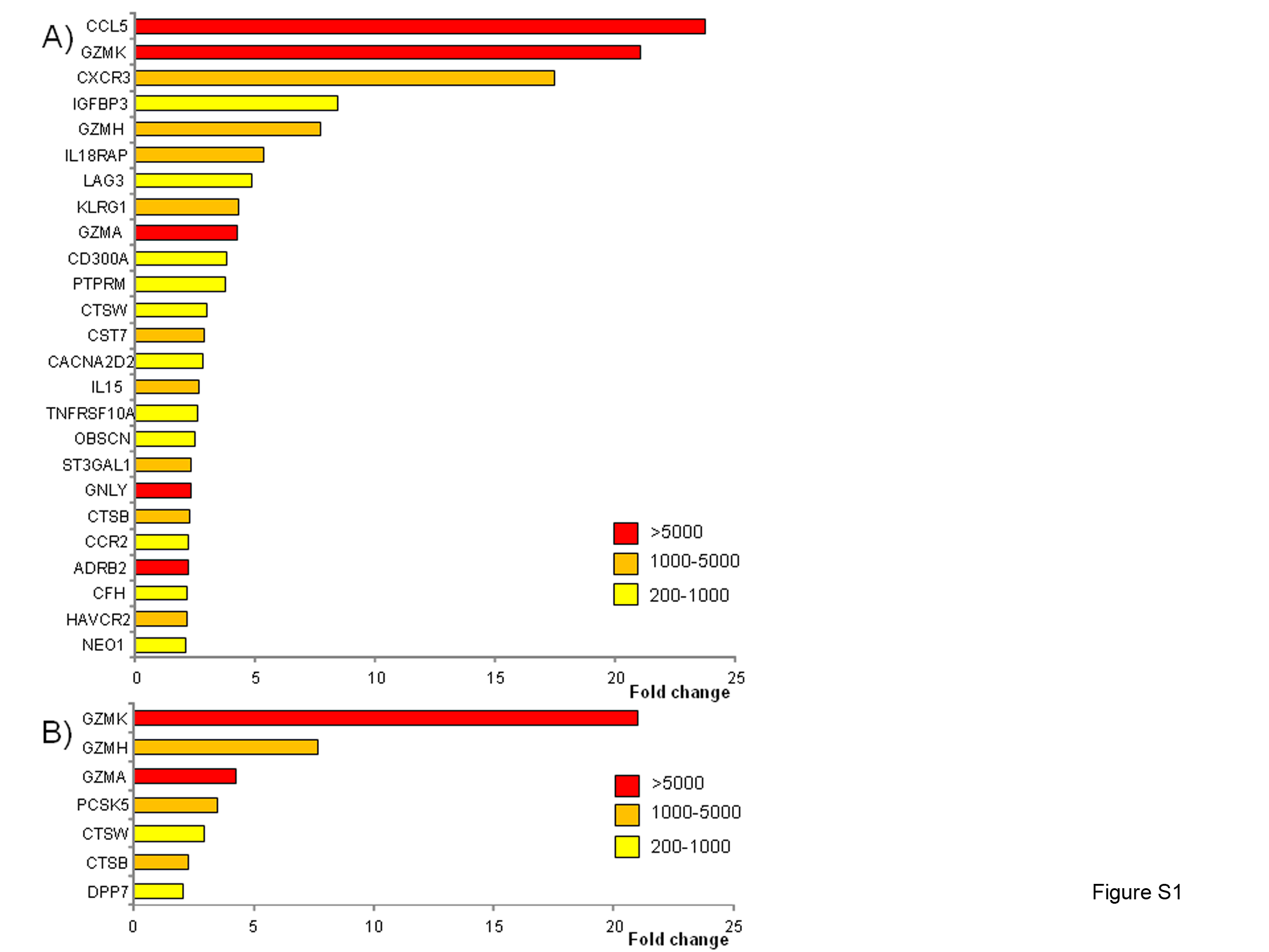

Supplement: Figure S1 — Fold change of Th1-dominant genes which belong to significantly over-represented functional categories. Over-representation analysis of Th1 dominant genes indicated that genes relevant to the “disulfide bond” category (p = 7.61E−05, p = 0.012 after Bonferroni correction) and “zymogen” category (p = 1.23E−04, p = 0.018 after Bonferroni correction) were significantly overrepresented. Figure S1 A and B indicate fold changed of Th1 which belong to “disulfide bond” and “zymogen” categories respectively. Th1 cell dominant genes Each bar represents a fold change of averaged signal intensity each gene in the Th1 microarray data divided by averaged signal intensity of the same gene in Th2 microarray data. Colors indicate the signal intensity of genes. Red: a very high expression level (>5000), orange: high level of expression (1000–5000), yellow: medium expression level (200–1000). (TIF) [file pone.0111405.s001.tif]
